# Hepatic Amyloidosis with an Extremely High Stiffness Value on Magnetic Resonance Elastography

**DOI:** 10.2463/mrms.ci.2015-0133

**Published:** 2016-03-21

**Authors:** Shuya MATSUDA, Utaroh MOTOSUGI, Ryo KATO, Masaru MURAOKA, Yuichiro SUZUKI, Mitsuharu SATO, Kuniaki SHINDO, Yasuhiro NAKAYAMA, Taisuke INOUE, Shinya MAEKAWA, Minoru SAKAMOTO, Nobuyuki ENOMOTO

**Affiliations:** 1 Department of Internal Medicine, University of Yamanashi, 1110 Shimokato, Chuo-shi, Yamanashi 409-3898, Japan; 2 Department of Radiology, University of Yamanashi; 3 Department of Internal Medicine, Kyonan Medical Center Fujikawa Hospital; 4 Department of Gastroenterology, Japan Community Healthcare Organization Yamanashi Hospital

**Keywords:** liver, amyloidosis, MR elastography

Hepatic amyloidosis, a rare disorder characterized by liver dysfunction, is not easy to diagnose clinically because it shares many clinical manifestations with other common chronic liver diseases. Magnetic resonance (MR) elastography is a novel technique for measuring the stiffness value of an object using MR imaging. An MR elastography is useful for the non-invasive diagnosis of liver fibrosis.^1,2^ However, some confounding factors besides fibrosis, such as inflammation, congestion, and portal hypertension, can increase liver stiffness values.^3^ In this report, we present a case of primary hepatic amyloidosis with an extremely high stiffness value on MR elastography, despite the lack of hepatic fibrosis. Amyloid deposition may be another confounding factor for MR elastography-based liver stiffness measurements, whereas an unexpectedly high liver stiffness value might indicate this rare liver disease.

A 63-year-old man was referred to our hospital because of anorexia and abnormal liver function. Physical examination revealed moderate ascites and a palpable hard liver 3 cm below the right costal margin. Laboratory tests demonstrated elevated aspartate aminotransferase (43 U/L), alanine aminotransferase (19 U/L), gamma-glutamyl transpeptidase (140 IU/L), alkaline phosphatase (853 IU/L), and total bilirubin (2.4 mg/dL) levels. Unenhanced computed tomography (CT) showed liver enlargement, particularly in the left and caudate lobes, and diffuse low attenuation. Splenomegaly and ascites were also present. Contrast-enhanced CT revealed narrowed hepatic veins. On MR imaging, signal intensities of liver parenchyma were slightly high on both T_2_WI and diffusion weighted imaging (DWI). No signal intensity loss was observed on T_1_ opposed-phase images. The R2 star value was ∼47.8/s, not increased, by multiecho two-dimensional (2D) gradient echo sequence. Gadoxetic acid-enhanced hepatobiliary phase images showed substantially reduced contrast agent accumulation in the liver, with a liver-to-spleen contrast ratio of 0.85 (Fig. 1). Liver stiffness measured using the MR elastography was 22 kPa (standard deviation, 3.5 kPa; minimum, 11 kPa; maximum, 33 kPa) (Fig. 2). Liver stiffness is typically ∼2 kPa for a healthy liver and 4–10 kPa for a cirrhotic liver. Therefore, this patient had markedly elevated liver stiffness.

Budd–Chiari syndrome was suspected, but transjugular venography revealed no hepatic venous occlusion. A transjugular liver biopsy was performed for pathological diagnosis. The pathological specimen showed an extracellular deposition of amorphous eosinophilic material along the sinusoidal wall. These deposits were positive for direct fast scarlet staining and negative for immunohistochemical serum amyloid A staining. The final pathological diagnosis was hepatic AL amyloidosis. Serum immunoelectrophoresis detected monoclonal IgA-κ protein. Chemotherapy including melphalan was not indicated because of progressive liver failure. Therefore, the patient was treated with high-dose dexamethasone. One month after diagnosis, the patient died of rapidly progressive liver failure despite treatment. Necropsy examination revealed multiple myelomas in bone marrow, and slight amyloid deposition in kidney, heart, and rectum.

The AL amyloidosis is a rare systemic disorder characterized by the extracellular deposition of fibrillar amyloid proteins derived from monoclonal immunoglobulin light chains. Hepatic amyloidosis is the hepatic manifestation of systemic amyloidosis. The prognosis of hepatic amyloidosis is poor, especially in patients with jaundice. Early diagnosis is critical for appropriate treatment. Some imaging features have previously been reported: hepatomegaly, poor parenchymal liver enhancement on contrast-enhanced computed tomography (CT)/MR imaging and prolonged T_1_ values in the liver. Recently, several articles have reported very high liver stiffness values in hepatic amyloidosis, as in our case, using ultrasound-based and MR-based elastography.^4^ Hepatic amyloidosis should be considered for cases of liver dysfunction with unexpectedly high liver stiffness values.

## Figures and Tables

**Fig. 1.**
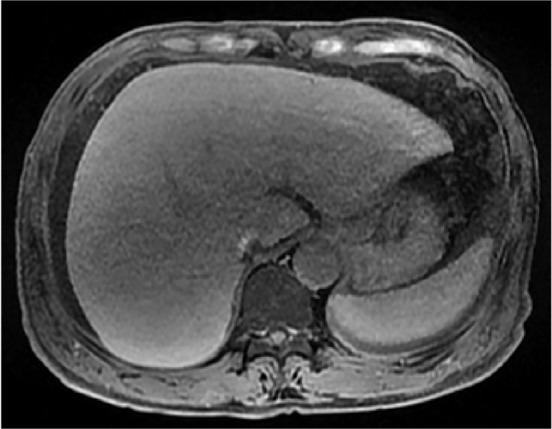
Gadoxetic acid-enhanced hepatobiliary phase images showed substantially reduced contrast agent accumulation in the liver, yielding a liver-to-spleen contrast ratio of 0.85.

**Fig. 2.**
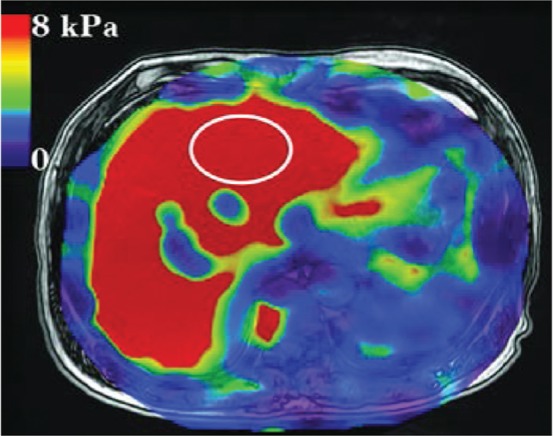
Magnetic resonance (MR) elastography stiffness map is fused on an opposed-phase T_1_-weighted gradient echo image. The liver stiffness measured using an MR elastography was 22 kPa (standard deviation, 3.5 kPa; minimum, 11 kPa; maximum, 33 kPa), indicating a marked elevation. The circle shows the region of interest for measuring the stiffness value.
